# Phase I clinical study of oral olaparib in pediatric patients with refractory solid tumors: study protocol

**DOI:** 10.1186/s12887-019-1409-7

**Published:** 2019-01-26

**Authors:** Masatoshi Takagi, Chitose Ogawa, Yuki Aoki-Nogami, Tomoko Iehara, Eri Ishibashi, Minoru Imai, Tetsuro Kihara, Kiyoshi Nobori, Kazuhisa Hasebe, Shuki Mizutani, Toshimi Kimura, Masashi Nagata, Masato Yasuhara, Kenichi Yoshimura, Pariko Yorozu, Hajime Hosoi, Ryuji Koike

**Affiliations:** 1 Department of Pediatrics and Developmental Biology, Tokyo Medical and Dental University (TMDU), Yushima 1-5-45, Bunkyo-ku, Tokyo, 113-8519 Japan; 2 Department of Pediatric Oncology, National Cancer Center, Tsukiji 5-1-1, Chuo-ku, Tokyo, 104-0045 Japan; 3 Department of Pediatrics, Kyoto Prefectural University of Medicine, Kawaramachi-Hirokoji, Kamigyo-ku, Kyoto, 602-8566 Japan; 4 University Research Administration Division, Tokyo Medical and Dental University (TMDU), Tokyo, Japan; 5 Medical Innovation Promotion Center, Tokyo Medical and Dental University (TMDU), Tokyo, Japan; 6 Department of Pharmacodynamics, Tokyo Women’s Medical University, Kawada-cho 8-1, Shinjuku-ku, Tokyo, 162-8666 Japan; 7 Department of Pharmacodynamics, Tokyo Medical and Dental University, Tokyo, Japan; 8 Innovative Clinical Research Center, Kanazawa University, Takara-machi 13-1, Kanazawa, Ishikawa 920-8641 Japan

**Keywords:** Olaparib, Phase I, Children, Solid tumor, Chemotherapy, Poly(ADP-ribose) polymerase

## Abstract

**Background:**

There is no established standard chemotherapy for recurrent pediatric solid tumors such as neuroblastoma and sarcoma. Since some of these tumor cells show dysfunctions in homologous recombination repair, the goal is to conduct a phase I study of olaparib, a poly(ADP-ribose) polymerase inhibitor. In this clinical trial, the aims are to evaluate the safety, tolerability, and efficacy of olaparib in pediatric patients with refractory solid tumors and to recommend a dose for phase II trials.

**Methods:**

In this open-label, multicenter study, olaparib tablets (62.5, 125, and 187.5 mg/m^2^ b.i.d.) will be administered orally in a standard 3 + 3 dose escalation design. Patients aged 3 to 18 years with recurrent pediatric solid tumors are eligible. Pharmacokinetic and pharmacodynamic analyses will also be performed.

**Discussion:**

This study aims to extend the indications for olaparib by assessing its safety and efficacy in pediatric refractory solid tumor patients.

**Trial registration:**

UMIN-CTR (UMIN000025521); Registered on January 4, 2017.

## Background

Childhood cancers develop in roughly in 1 of 6000–6500 children and adolescents under age 20 years. Approximately 40–50% of childhood cancers are hematological malignancies, followed by brain tumors. Among other solid tumors, two-thirds consist of neuroblastoma, hepatoblastoma, nephroblastoma, and germ cell tumors, and one-third consist of sarcomas such as rhabdomyosarcoma, Ewing’s sarcoma, and osteosarcoma (Table 1) [1]. About 1000–1500 pediatric solid tumors develop annually in Japan. Although most pediatric tumors are curable, some are refractory. For example, in the case of neuroblastoma, the survival rate of low- to intermediate-risk cases is 90%, while that of high-risk cases is approximately 30%.

**Table 1:** Treatment results of representative pediatric solid tumors after the first relapse

Tumor types	Number of cases	MST (months)	OS		Ref.
			%	Year	
Neuroblastoma	2266	–	20	5	[22]
	357	10	14	10	[23]
Hepatoblastoma	59	12	43	3	[24]
Rhabdomyosarcoma	605	10	17	5	[25]
	125	15	28	5	[26]
Ewing’s sarcoma	107	12	19	5	[27]
	55	–	23	5	[28]
Osteosarcoma	43	15	35	3	[29]
Nephroblastoma	170	–	48	5	[30]

*MST* median survival time, *OS* overall survival

Due to the rarity of pediatric tumors, a randomized, phase III clinical trial using a newly developed drug is difficult to design, especially for refractory cases. The efficacy of already established standard chemotherapy in these tumors is limited. In addition, the response rate to second-line chemotherapy is less than 50%, and the prognosis of recurrent pediatric solid tumors is very poor (Table 1). These situations have prompted us to develop a novel therapeutic agent for refractory or recurrent pediatric solid tumors.

In neuroblastoma, *MYCN* amplification is a well-characterized genetic alteration that correlates directly with advanced stage and a poor prognosis. Loss of 1p, 3p, and 11q is also observed in advanced neuroblastomas and is associated with an unfavorable prognosis [2, 3]. Genomic alterations, such as loss and single nucleotide variants, in the *ATM* gene and other DNA damage response (DDR)-associated genes were found in nearly half of neuroblastoma and neuroblastoma-derived cell lines, particularly in advanced stages [4]. ATM-defective cells are known to exhibit dysfunctions in homologous recombination repair, suggesting a potential for synthetic lethality by a poly(ADP-ribose) polymerase (PARP) inhibitor. Indeed, 83.3% of neuroblastoma-derived cell lines showed sensitivity to PARP inhibition [4]. With a full complement of repair pathways, normal cells can compensate for the loss of individual DDR pathways, such as PARP inhibition. However, loss of one or more DDR pathway(s) in response to oncogenic stress can leave tumor cells vulnerable to PARP inhibition and induce cancer-specific cell death through the process of synthetic lethality.

Ewing’s sarcoma cells exhibit high levels of DNA damage and similarity in phenotype to *BRCA1/2* mutant breast cancer, providing a molecular basis for the high sensitivity of Ewing’s sarcoma to PARP1 inhibitors [5, 6]. More than 80% of osteosarcomas show a specific combination of single-base substitutions, LOH, or large-scale genome instability signatures characteristic of BRCA1/2-deficient tumors, indicating a BRCAness phenotype [7]. It has also been shown that osteosarcoma cells with genetic signatures of BRCAness are susceptible to the PARP inhibitor [8]. These results suggest that a PARP inhibitor may be an effective drug for Ewing’s sarcoma and osteosarcoma.

A PARP inhibitor, olaparib, is widely and safely used not only for BRCA1/2-deficient breast and ovarian cancer patients, but also for many other adult cancer patients [9–13]. Thus, there is a high possibility that olaparib would be effective for pediatric solid tumors. In this study, the aim is to develop a therapeutic approach using olaparib in pediatric patients with refractory solid tumors, such as neuroblastoma and sarcomas.

## Methods/design

### Objectives

The objectives are to evaluate safety and tolerability of oral olaparib in pediatric patients with refractory solid tumors to determine dose-limiting toxicity (DLT) and a recommended dose (RD) for subsequent phase II clinical studies.

### Study design

This study is the first phase I, multicenter (Tokyo Medical and Dental University, National Cancer Center Hospital, and Kyoto Prefectural University of Medicine), single-arm, open-label trial of olaparib in pediatric patients with refractory solid tumors. The protocol has been reviewed and approved by the Institutional Review Boards of each participating institution (Tokyo Medical and Dental University: Approved No. 2016–1001, National Cancer Center: Approved No. T4406 and Kyoto Prefectural University of Medicine: Approved No. 2017–036).

### End points

1. Primary endpoint
  Incidence of DLT
2. Secondary endpoint
  i) Incidence and type of adverse events
  ii) Analysis of pharmacokinetics of orally administered olaparib
3. Exploratory endpoint
  i) Response rate of each tumor type
  ii) Analysis of pharmacodynamics monitored by PARP activity in peripheral blood mononuclear cells

### Inclusion criteria

All of the key criteria listed below are required for inclusion.

1. Patients and/or their representatives must provide written, informed consent for this clinical study.
2. Patients aged 3 to 18 years.
3. Pathologically confirmed pediatric refractory solid tumors described in the International Pediatric Cancer Classification, Third edition, group IV-XII, excluding hematopoietic tumors and primary central nervous system tumors [1]. Refractory tumors are defined as resistant to more than two types of chemotherapy regimens.
4. One or both of the following are fulfilled.
  i) Tumors are confirmed by computed tomography (CT) or magnetic resonance imaging (MRI).
  ii) Tumor cells are confirmed by cytology or bone marrow examination.
5. The patient is expected to survive for 4 months or more after the administration of investigational drug.
6. The function of each organ and bone marrow is normal within 14 days before registration according to the following criteria.
  i) Hemoglobin ≥8.0 g/dL without packed red blood cell transfusion within 28 days before enrollment.
  ii) Leukocyte count ≥3000/μL and neutrophil count ≥1500/μL without administration of granulocyte-colony stimulating factor (G-CSF) within 14 days or administration of polyethylene glycol (PEG)-conjugated G-CSF within 21 days before registration.
  iii) Platelet count ≥100,000/μL without platelet concentrate transfusion within 14 days before enrollment.
  iv) Exclusion of myelodysplastic syndrome or acute leukemia by peripheral blood smear specimens.
  v) Total bilirubin level ≤ 1.5 × upper limit of normal (ULN)
  vi) AST and ALT ≤2.5 × ULN (or ≤ 5.0 × ULN in hepatic tumor or hepatic metastasis patients)
  vii) Serum creatinine level ≤ 1.5 × ULN
7. Performance scale: Lansky play-performance scale (under 16 years of age) or Karnofsky scale (16 years of age and over) over 70.
8. Not pregnant. Pregnancy test is negative by urine or serum test within 28 days before registration.
9. Patient can take a tablet with a diameter of 6 mm.

### Exclusion criteria

Patients are excluded from enrollment if they meet any of the key criteria listed below.

1. Patients related to the planning and implementation of this clinical trial.
2. Patients who have been enrolled in this clinical trial and received the investigational drug.
3. Patients who have undergone administration of olaparib or other PARP inhibitors.
4. Patients with types of malignant tumors other than the original tumor.
5. Patients who have received the final treatment of systemic chemotherapy or radiotherapy (except for the purpose of palliative treatment) within 21 days before enrollment.
6. Patients who have brain metastases with uncontrollable symptoms. However, imaging diagnosis is not necessary to confirm exclusion of brain metastasis. If corticosteroid administration is initiated more than 28 days prior to enrollment, it may be administered at a fixed dose prior to or during the trial period. If patients have a spinal cord tumor, the patient should receive treatment that makes symptoms stable for 28 days. Combined use of radiation therapy to control symptoms is not permitted.
7. Patients with gastrointestinal disorders that may interfere with the absorption of investigational drug.
8. The body surface area (BSA) at registration is less than 0.40 m^2^.
9. Patients who have been given other investigational drugs within 21 days prior to enrollment.
10. CYP3A4 inhibitor cannot be stopped 14 days before the start of investigational drug administration.
11. CYP3A4 inducer cannot be stopped 21 days before the start of investigational drug administration.
12. Pretreatment toxicity, common terminology criteria for adverse events (CTCAE) version 4.0 grade 2 or higher, except for stable alopecia, onychomadesis, and hearing disorder, occurs.
13. Patients who had major surgery (laparotomy, thoracotomy, craniotomy, etc.) within 14 days before registration and have not recovered from its effects.
14. Patients with severe and uncontrollable diseases, or active infection, as shown below.
  i) QT extension, QTc > 470 msec, is observed twice or more within 24 h before enrollment, or familial long QT syndrome.
  ii) Uncontrollable ventricular arrhythmia
  iii) Respiratory failure, SpO _2_ < 94% indoors
  iv) Pulmonary diseases such as bilateral interstitial pneumonia and obstructive bronchitis
  v) Uncontrollable active infection with drug treatment
  vi) Psychiatric disorder with poor control
15. Breastfeeding during the trial period is inevitable.
16. Immunodeficient condition such as serologically HIV positive or receiving antiviral therapy.
17. Patients with active hepatic diseases, such as HBs antigen- or HCV antibody-positive.
18. Hypersensitivity to olaparib or olaparib tablet excipients.
19. Patients who received autologous hematopoietic stem cell transplantation within 112 days, 4 months, before enrollment.
20. Patients who received allogenic hematopoietic cell transplantation.
21. Patients judged inappropriate to participate in the study for any other reason by the investigator.

### Study drug

The investigational drug is olaparib, the identification code is AZD2281, and the chemical name is 4-[(3-{[4-(cyclopropylcarbonyl)piperazin-1-yl]carbonyl}-4-fluorophenyl)methyl]phthalazin-1(2H)-one. The agent type is a tablet, inclusion volume is 25 mg per tablet, and drugs are stored at 30 °C or less. Olaparib is manufactured by AbbVie Inc. (North Chicago, IL) and provided by Astrazeneca (Cambridge, United Kingdom), and trade name is Lynparza®. It is an inhibitor of PARP, an enzyme involved in DNA repair. It acts against cancers in patients with hereditary BRCA1 or BRCA2 mutations, which include some ovarian, breast, and prostate cancers [9–12].

### Protocol treatment

This is the first phase I clinical study of olaparib in pediatric patients. In adults, the olaparib tablet was shown to be well tolerated up to the 300 mg b.i.d. dose in non-Japanese, as well as in Japanese, patients with solid tumors [14, 15]. The present study is, therefore, designed to evaluate the safety and tolerability of olaparib at 100, 200, and 300 mg, which are one-third, two-thirds, and the same doses as the adult dose, respectively, in the previous study (300 mg), and to determine the DLT of olaparib in order to obtain the basis for RD for the next phase.

It has been reported that children can receive the same weight-based or BSA-based doses as adults in many cases [16]. The standard Japanese adult BSA is 1.6 m^2^, with the calculation formula of BSA as follows:

$$ \mathrm{BSA}\ \left({\mathrm{m}}^2\right)=\surd \left(\left(\mathrm{height}\left(\mathrm{cm}\right)\times \mathrm{weight}\left(\mathrm{kg}\right)\right)\div 3600\right) $$

(The height is rounded off to the nearest whole number, the weight is rounded off to the first decimal place, and the BSA is rounded off to the second decimal place).

Thus, the well-tolerated dose of 300 mg in adults converted using BSA is 187.5 mg/m^2^ per dose. Similarly, 200 mg is 125 mg/m^2^, and 100 mg is 62.5 mg/m^2^. The clinical hypothesis is that single agent administration of olaparib 187.5 mg/m^2^ b.i.d. to pediatric refractory solid tumor patients can be performed safely. Since patients take 25 mg tablets, the one-time dose is determined according to BSA as shown in Table 2.

**Table 2:** Olaparib administration doses (mg) per BSA

BSA (m^2^)	Olaparib administration doses (mg b.i.d.)		
	1st dose (62.5 mg/m^2^)	2nd dose (125 mg/m^2^)	3rd dose (187.5 mg/m^2^)
0.40 ≤ BSA < 0.50	25	50	75
0.50 ≤ BSA < 0.60	25	50	75
0.60 ≤ BSA < 0.70	25	75	100
0.70 ≤ BSA < 0.80	25	75	125
0.80 ≤ BSA < 0. 90	50	100	150
0.90 ≤ BSA < 1.00	50	100	150
1.00 ≤ BSA < 1.10	50	125	175
1.10 ≤ BSA < 1.20	50	125	200
1.20 ≤ BSA < 1.30	75	150	225
1.30 ≤ BSA < 1.40	75	150	225
1.40 ≤ BSA < 1.50	75	175	250
1.50 ≤ BSA < 1.60	75	175	275
1.60 ≤ BSA	100	200	300

Only on the first day (cycle 0 day 1: C0d1), the patient takes olaparib once in the morning 1 h after a meal and fasts for 2 h after administration to avoid the effect of meals. The patient is observed for 48 h for the pharmacokinetic and pharmacodynamic analyses. From C0d1 evening to C0d3 evening, the patient is not administered the investigational drug. Cycle 1 starts from the fourth day of cycle 0, and the patient is administered the drug in the morning and evening, every 12 h, for 28 days as one cycle.

Patients who continue to benefit from treatment, that is, show complete response (CR), partial response (PR), or stable disease (SD), may have the option to continue treatment upon agreement between the investigator and sponsor, and upon study drug availability. If treatment continues beyond the predesigned schedule, study procedures should continue to be performed at the same frequency described in the dose escalation phase.

### Definition of DLT

DLT is evaluated by the standard 3 + 3 dose escalation design. The DLT evaluation period is from the first day of cycle 0 to the 28th day of cycle 1 (C0d1 - C1d28), including the drug holiday. In case of discontinuation of the investigational drug beyond C1d28 due to toxicity related to the drug, the DLT evaluation period is extended up to 14 days.

DLT is defined as the following events occurred during the DLT evaluation period and is judged by the investigator or sub-investigator as having a high probability of investigational drug relevance, with or without disappearance of toxicity.

1. Neutropenia (< 500/μL) that persists for more than 5 days without fever.
2. Neutropenia (< 500/μL) with fever or sepsis
3. Thrombocytopenia (< 25,000/μL)
4. CTCAE grade 3 or higher anemia
5. When blood transfusion is performed.
6. CTCAE grade 3 or 4 non-hematologic toxicity, except for fatigue, nausea, vomiting, diarrhea, muscle pain, and arthralgia recovering to CTCAE grade 2 or less within 7 days after treatment.
7. CTCAE grade 2 or higher cardiotoxicity or neurotoxicity
8. Toxicity resulting in discontinuation of protocol treatment during the first cycle.
9. Other toxicity not recovering to grade 1 or less within 14 days of events that resulted in drug withdrawal during the first cycle. When the drug is taken only once a day, it is defined as 1 day off.

Determination of the existence of DLT or undecidable is primarily performed by the investigator at each institution, but the final judgment is made by the coordinating doctor. In case of doubt in the judgment of DLT, opinions can be requested from the efficacy and safety evaluation committee.

### Standard 3 + 3 dose escalation for DLT evaluation and RD definition

The daily first, second, and third doses of olaparib are 125, 250 and 375 mg/m^2^, respectively. Dose escalation is performed in a standard phase I 3 + 3 design. The target sample size is 18. A minimum of 6 cases are required for DLT evaluation, and the dose level showing DLT in 1 or less of 6 patients is judged as the RD. The RD is determined by the clinical trial coordinating doctor after deliberation with the efficacy and safety evaluation committee.

### Pharmacokinetics of olaparib in pediatric patients

In a pharmacokinetic analysis, the plasma concentration of olaparib is measured before the first dose, and 1, 2, 3, 6, 8, and 12 h (24 and 48 h only in C0d1) after administration on C0d1 and C1d15. Pharmacokinetic parameters, such as AUC, C_max_, T_max_, and T_1/2_, will be estimated.

### Pharmacodynamics of olaparib in pediatric patients

Blood samples are collected 6 h before and 6 h after the administration of olaparib on C0d1, and 6 h before and 6 h after the first dose of C1d15. PARP inhibitory activity by olaparib is measured in peripheral blood mononuclear cells. When the protocol treatment is interrupted or original diseases are confirmed to have exacerbated, the PARP inhibitory activity in blood mononuclear cells should be measured.

### Efficacy assessment method

Tumor reduction effect is assessed according to new response evaluation criteria in solid tumors: Revised RECIST guideline, version 1.1 [17]. Radiologic assessments using CT and/or MRI are performed within 28 days before registration, which is used as baseline, and at odd cycles. At each time point, the treatment response is assessed as CR, PR, progressive disease (PD), SD, or not all evaluated (NE). Overall response at each time point is also assessed according to revised RECIST.

### Statistical methods

Descriptive statistics are used to define the study population, safety, tolerability, pharmacokinetic and pharmacodynamic data, and tumor response.

## Discussion

One of the hallmarks of cancer is genomic instability, which is associated with clonal evolution. Historically, cancer therapy is targeted to induce DNA damage to kill cancer cells by irradiation or chemotherapy. Recently, molecular-targeted therapy focusing on cancer-specific molecular signatures has been developing, and most are inhibitors of signaling pathways. Molecular-targeted therapy based on inhibiting DDR in cancer cells offers the potential for a greater therapeutic window by tailoring treatment to patients with tumors lacking specific DDR functions. The PARP inhibitor is one of a new class in this field. The best-known disease-associated examples of defective components of homologous recombination repair are the breast- and ovarian-associated tumor suppressor genes *BRCA1* and *BRCA2* [9–12]. The recent approval of olaparib for treating tumors harboring *BRCA1* or *BRCA2* mutations represents the first drug based on this principle. Various factors other than BRCA1 and BRCA2, such as ATM, are involved in the homologous recombination repair process. Several cancers have mutations in or epigenetically silenced homologous recombination-associated genes, which explains the genetic instability that drives cancer development. In the pediatric cancer field, inactivation of these genes has been reported in neuroblastoma, Ewing’s sarcoma, and osteosarcoma [4–8, 18]. We, therefore, have designed a phase I clinical study using olaparib, a PARP inhibitor, for these refractory solid tumors in pediatric patients.

Development of new drugs specifically for pediatric cancers is scarce because of the small numbers of patients, limitations by regulations for pediatric drugs, and insufficient return on investment. Therefore, children have usually been excluded from first clinical trials of promising new cancer drugs, possibly resulting in inappropriate use of new drugs without enough information in children and even low survival rates based on inadequate existing treatment options. Phase I trials of new drugs in children are generally carried out only after several trials in adults [19]. Furthermore, these clinical trials are mostly initiated by academic investigators. These situations delay the design of phase I clinical trials in children.

Pediatric and adult patients may have different toxicities for some drugs [19–21]. Younger children may be at risk for developmental toxicities with certain cancer drugs that would not have been identified in adults, and longer survival times of children can be associated with possible later side effects such as secondary cancers. Therefore, new cancer drugs must generally be validated in pediatric populations.

Although the molecular signatures of pediatric and adult cancers are different, there are several common pathways that are appropriately targeted by drugs used in adults. The PARP inhibitor olaparib is one of them. Thus, this study aims to extend the indications of olaparib by assessing its safety and efficacy in pediatric refractory solid tumor patients.

## Abbreviations

BSA: Body surface area
CR: Complete response
CTCAE: Common terminology criteria for adverse events
DDR: DNA damage response
DLT: Dose-limiting toxicity
G-CSF: Granulocyte-colony stimulating factor
NE: Not all evaluated
PARP: Poly(ADP-ribose) polymerase
PD: Progressive disease
PEG: Polyethylene glycol
PR: Partial response
RD: Recommended dose
RECIST: Response Evaluation Criteria in Solid Tumours
SD: Stable disease
ULN: Upper limit of normal
